# A Case Report of Secondary Spontaneous Pneumomediastinum Induced by Vaping

**DOI:** 10.7759/cureus.35153

**Published:** 2023-02-18

**Authors:** Michael Kartiko, Aisha Miller

**Affiliations:** 1 Internal Medicine, Piedmont Macon Medical Center, Macon, USA; 2 Radiology, Creighton University School of Medicine, Omaha, USA

**Keywords:** pulmonary barotrauma, e-cigarette smoking, e-cigarette, pneumomediastinum, effects of vaping, e-cigarette or vaping use-associated lung injury (evali), spontaneous pneumomediastinum (spm)

## Abstract

We present a rare case of vaping-induced spontaneous pneumomediastinum in a young healthy female. The patient presented to the emergency department with the chief complaint of acute onset chest pain. Imaging studies, chest X-ray, and computed tomography of the chest showed findings of pneumomediastinum. The patient was counseled on vaping cessation and discharged after 48 hours.

## Introduction

Pneumomediastinum is a rare pathological condition characterized by free air in the mediastinal space. Spontaneous or primary pneumomediastinum occurs without any apparent cause. Some causes of a secondary pneumomediastinum include asthma, chronic obstructive pulmonary disease (COPD) exacerbations, esophageal rupture, iatrogenic damage, or trauma [1]. In this report, we present a rare case of spontaneous pneumomediastinum in a young, healthy female with a social history significant for years of daily vaping and no other past medical history. The patient presented with a sudden onset of severe throat pain, odynophagia, and chest pain. Imaging studies, chest X-ray, and CT scan of the chest showed findings of pneumomediastinum. The patient was admitted for 48 hours and counseled on cessation of vaping.

## Case presentation

**Figure 1:**
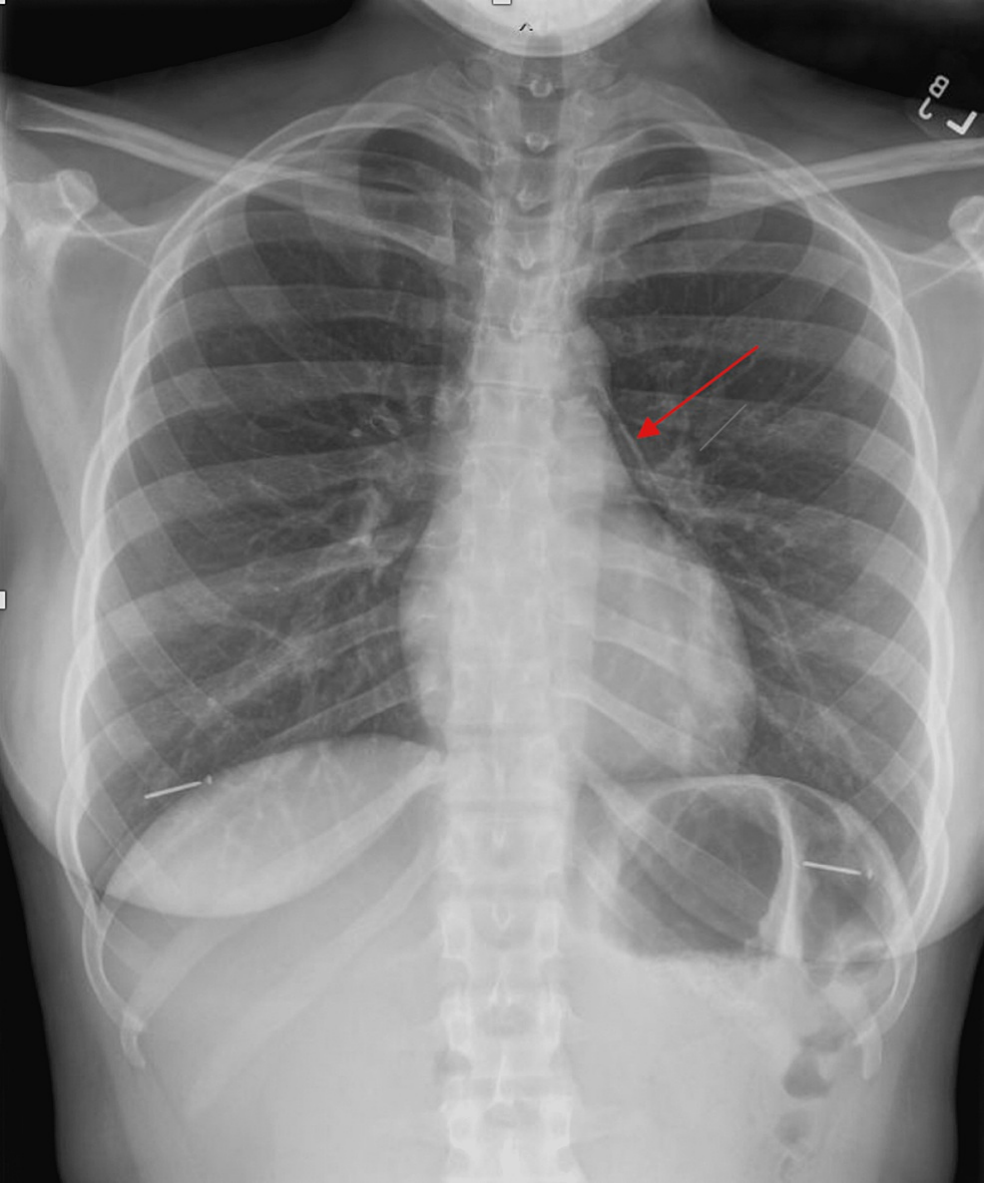
Chest X-ray showing pneumomediastinum.

**Figure 2:**
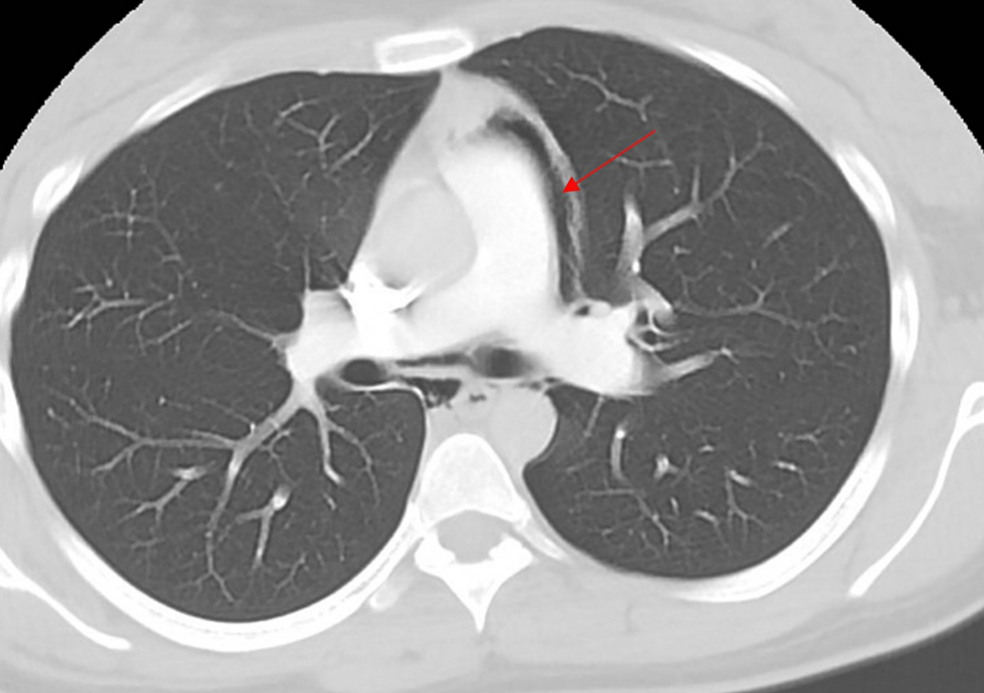
Axial computerized tomography of the chest showing pneumomediastinum

**Figure 3:**
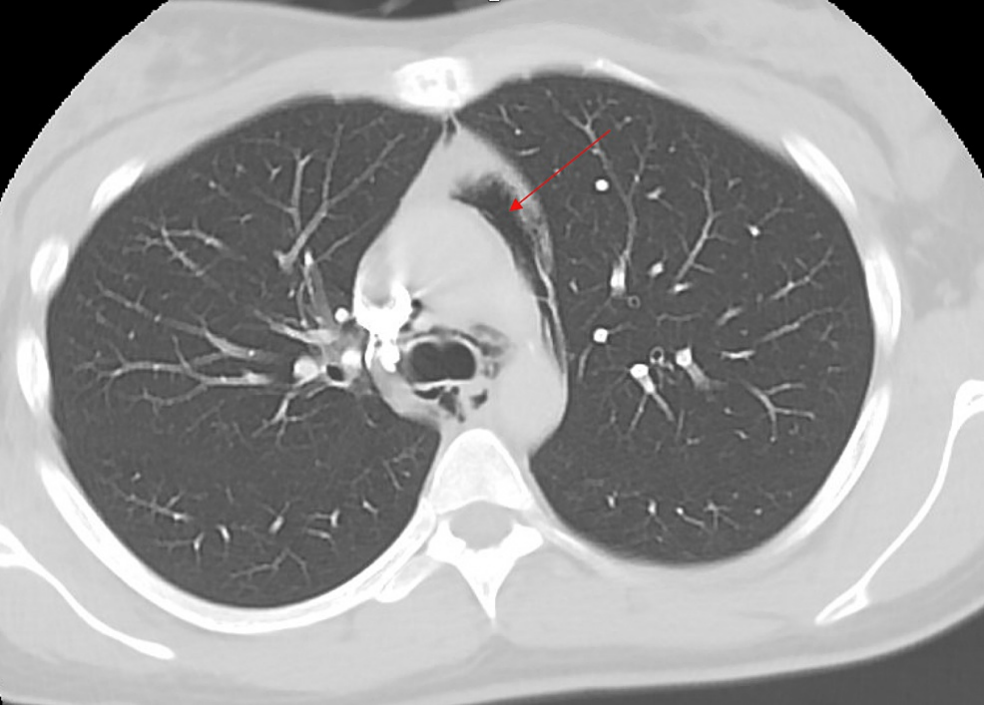
Axial computerized tomography of the chest showing pneumomediastinum.

**Figure 4:**
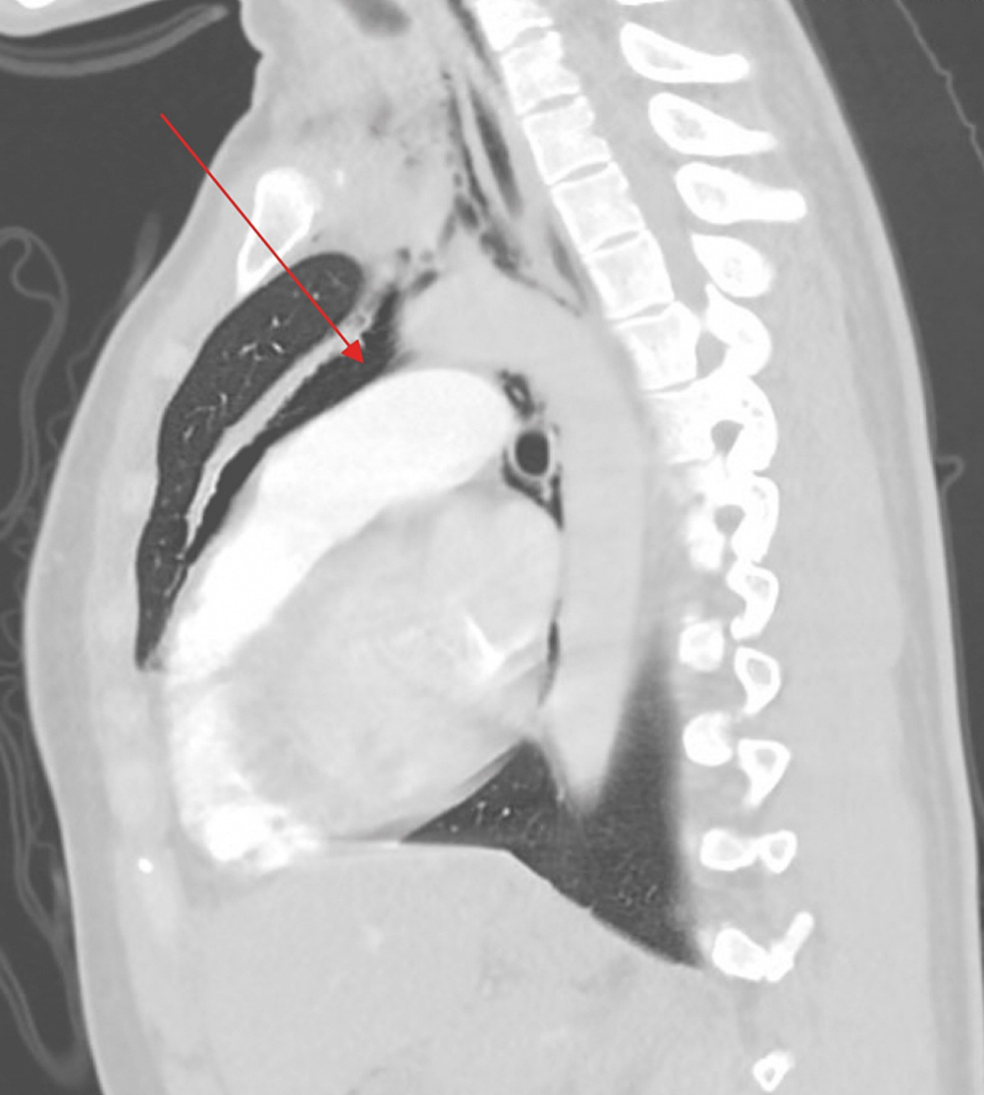
Sagittal computerized tomography of the chest showing pneumomediastinum.

**Figure 5:**
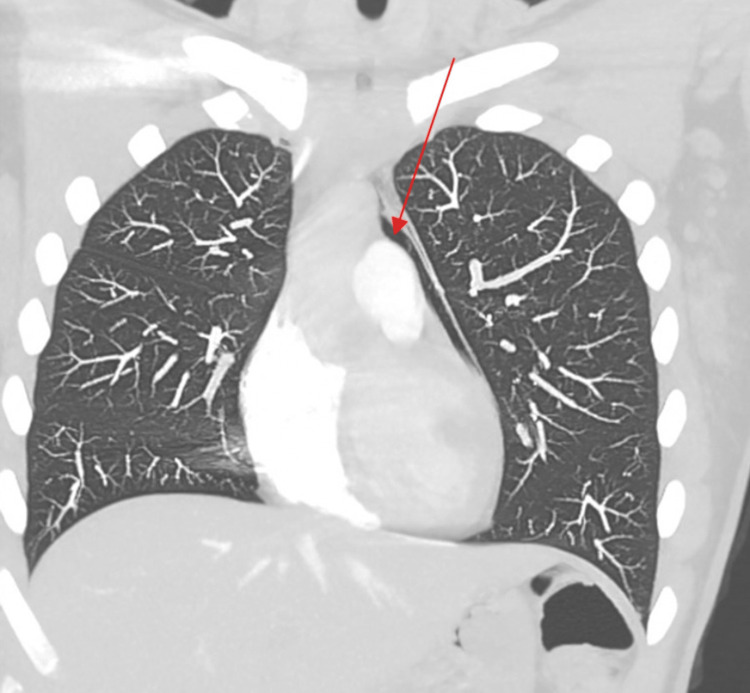
Maximum-intensity-projection image of the chest showing pneumomediastinum.

A 26-year-old African American female with a two-year history of daily vaping and no other medical history presented to the emergency department with sudden onset severe throat pain with associated odynophagia and sharp non-radiating anterior chest pain. Her symptoms were exacerbated by deep breathing with no relieving factors. She was at rest when the pain abruptly started and became progressively worse. She denied any dyspnea, nausea, vomiting, coughing, fever, or chills. Her social history was significant for regular daily electronic cigarette vaping for the past two years and no cigarette smoking or recreational drug use. On the initial presentation, she was tachycardic and borderline tachypneic. Chest X-ray (Figure 1) and a CT angiogram of the chest (Figures 2-5) showed findings of a small to moderate sized pneumomediastinum along the left heart border, tracking upwards along the aortic arch without pneumothorax.

The patient was admitted for observation with proper pain control and instructed to withhold food and fluids by mouth. A fluoroscopic esophagram showed no evidence of esophageal perforation or leak. A cardiothoracic surgeon and pulmonologist evaluated the patient with recommendations to continue supportive care and monitor the patient with serial chest X-rays with no other intervention. Twenty-four hours after admission, chest imaging demonstrated the decreased size of the pneumomediastinum. The patient was counseled in detail on vaping cessation and discharged home after 48 hours.

## Discussion

The source of air extravasation in pneumomediastinum is most commonly identified as either the tracheobronchial tree or the esophagus. Spontaneous or primary pneumomediastinum occurs with no apparent cause. There are many causes of secondary pneumomediastinum, including asthma or COPD exacerbation, esophageal rupture (Boerhaave Syndrome), iatrogenic causes (intubation, endoscopy, thoracotomy), or traumatic causes (blunt or penetrating) [1]. In this case, it was suspected that the patient’s spontaneous pneumomediastinum occurred secondary to constant daily vaping. The inhalation of illicit substances, especially when done repeatedly and forcefully, leads to a sudden increase in intra-alveolar pressure resulting in barotrauma that causes alveolar rupture. Alveolar rupture causes air to dissect into the peribronchial and perivascular space and ultimately into the mediastinum [1-3]. By a similar mechanism, alveolar rupture secondary to vaping can also cause pneumothorax. There have been several cases of vaping associated spontaneous pneumothorax (VASP) reported in the literature [4]. There have also been reported cases of spontaneous pneumomediastinum in young patients caused by inhalation of marijuana [2,5] and crack cocaine [6,7]. Vaping-induced pneumomediastinum is a less commonly reported phenomenon, with only four other cases reported in the literature [3,8-10]. Each reported case presented similarly to ours in an otherwise young, healthy patient with no known past medical history.

We suspect that the adverse effects of vaping will continue to become more prevalent as electronic cigarettes have been widely marketed and accepted as a safer alternative to conventional cigarette smoking, particularly among younger individuals [6,11-13]. There have been many reported cases of e-cigarette or vaping-associated lung injury (EVALI), ranging from those managed in an outpatient setting to those resulting in life-threatening hypoxic respiratory failure requiring ICU level of care [13]. While it is widely accepted that conventional cigarette smoking poses a multitude of adverse health effects, there is still much less data on the adverse effects of electronic cigarette use [11,12]. In the majority of cases, spontaneous pneumomediastinum is a self-limiting condition requiring only supportive care with proper pain control, antitussives, and imaging studies to confirm resolution. Patients are typically hemodynamically stable and can be admitted for short inpatient observation [2,3,5,7-10]. The diagnosis of spontaneous pneumomediastinum is largely one of exclusion and is confirmed with imaging.

In our case, no elements of the patient’s history suggested secondary causes of pneumomediastinum. Nevertheless, the esophageal rupture was first ruled out with a fluoroscopic esophagram. Bronchoscopy was also briefly considered to rule out the tracheobronchial tree as a potential source. However, the patient remained stable and a chest X-ray obtained one day after admission showed improvement in the size of the pneumomediastinum, therefore, a more conservative management approach was appropriate. When evaluating a young, otherwise healthy patient presenting with chest pain, it is crucial to obtain a detailed social history and consider pneumomediastinum as a diagnosis on the differential.

## Conclusions

Healthcare providers should be aware of the various presentations of vaping-associated lung injury. It is a common misconception that vaping is a safer alternative to traditional cigarette smoking and carries minimal health risks, which may cause patients to vape heavily or utilize vaping as a means to quit smoking. It is important to note that when obtaining a social history, a patient may specifically deny conventional cigarette use but may be a regular user of electronic cigarettes, which, as we demonstrate in this case, is not without its own adverse risks. Providers should specifically inquire about history of vaping and the use of electronic cigarettes when obtaining a social history. Counseling patients on the risks of vaping and encouraging abstinence from inhalation of any type of illicit substance is important to prevent vaping-induced lung injuries. 

## References

[1] Iteen AJ, Bianchi W, Sharman T (2022). Pneumomediastinum. https://www.ncbi.nlm.nih.gov/books/NBK557440/.

[2] Puri C, Rhee K, Harish VK, Slack D (2021). Marijuana induced spontaneous pneumomediastinum. J Community Hosp Intern Med Perspect.

[3] Adhikari R, Manduva D, Malayala SV, Singh R, Jain NK, Deepika K, Koritala T (2021). A rare case of vaping-induced spontaneous pneumomediastinum. Cureus.

[4] Ashraf O, Nasrullah A, Karna R, Alhajhusain A (2021). Vaping associated spontaneous pneumothorax - a case series of an enigmatic entity!. Respir Med Case Rep.

[5] Weiss ZF, Gore S, Foderaro A (2019). Pneumomediastinum in marijuana users: a retrospective review of 14 cases. BMJ Open Respir Res.

[6] Cao DJ, Aldy K, Hsu S, McGetrick M, Verbeck G, De Silva I, Feng SY (2020). Review of health consequences of electronic cigarettes and the outbreak of electronic cigarette, or vaping, product use-associated lung injury. J Med Toxicol.

[7] Macrae C, Brown C, Aiken C, Jamdar R (2019). Pneumomediastinum as a complication of cocaine abuse. Clin Med (Lond).

[8] Alam MD, Hussain K, Garedew S, Imtiaz M (2021). Vaping and commitment flu-B infection is a deadly combination for spontaneous pneumomediastinum. Case Rep Pulmonol.

[9] Burgwardt S, Huskic A, Schwartz G, Mason DP, Tapias L, Podgaetz E (2020). Spontaneous pneumomediastinum secondary to electronic cigarette use. Proc (Bayl Univ Med Cent).

[10] Marasco RD, Loizzi D, Ardò NP, Fatone FN, Sollitto F (2018). Spontaneous pneumomediastinum after electronic cigarette use. Ann Thorac Surg.

[11] Kalininskiy A, Bach CT, Nacca NE (2019). E-cigarette, or vaping, product use associated lung injury (EVALI): case series and diagnostic approach. Lancet Respir Med.

[12] Marques P, Piqueras L, Sanz MJ (2021). An updated overview of e-cigarette impact on human health. Respir Res.

[13] Jose T, Hays JT, Warner DO (2020). Improved documentation of electronic cigarette use in an electronic health record. Int J Environ Res Public Health.

